# Ultrastructural changes of smooth and rough titanium implant surfaces induced by metal and plastic periodontal probes

**DOI:** 10.1007/s00784-020-03341-1

**Published:** 2020-06-21

**Authors:** Matthias Folwaczny, Torsten Rudolf, Iris Frasheri, Madlena Betthäuser

**Affiliations:** 1 Department of Conservative Dentistry and Periodontology, University Hospital, Ludwig-Maximilians-University, Goethestr. 70, D-80336 Munich, Germany; 2 ZEISS Microscopy Customer Center Europe, Carl Zeiss Microscopy GmbH, Oberkochen, Germany

**Keywords:** Topography, Ultrastructure, Confocal, Implant, Probing

## Abstract

**Objectives:**

To determine the ultrastructural changes of titanium surfaces of dental implants induced by the tip of periodontal probes.

**Materials and methods:**

A total of 40 samples of smooth and rough surfaces of titanium implants were randomly assigned for the treatment with metal or plastic periodontal probes under application angles of 20° and 60°. Titanium surfaces have been evaluated with CLSM prior and following to experimental probing determining various standardized 2D and 3D roughness parameters.

**Results:**

The average profile and surface roughness (Ra and Sa) showed no significant difference between treated and untreated samples on smooth and rough surface areas irrespective of the probe material. On smooth surfaces several amplitude roughness parameters were increased with metal probes but reached significance only for Rp (*p* = 0.007). Rough surface parts showed a slight but not significant reduction of roughness following to the contact with metal probes. The surface roughness remained almost unchanged on smooth and rough implant surfaces using plastic probes. The surface roughness on implant surfaces was not dependent on the application angle irrespective of the probe material.

**Conclusion:**

Probing of titanium implants with metal probes and even less with plastic probes causes only minor changes of the surface roughness. The clinical significance of these changes remains to be elucidated.

**Clinical relevance:**

Using plastic probes for the clinical evaluation of the peri-implant sulcus might avoid ultrastructural changes to titanium implant surfaces.

## Introduction

Current evidence suggests a rather high prevalence of inflammatory conditions around dental implants. According to a recent review, peri-implant mucositis occurs in around half of the implants [1]. If not recognized and treated, this entity can progress to peri-implantitis which affects up to one fourth of dental implants [2, 3]. In contrast to peri-implant mucositis, peri-implantitis leads to the loss of crestal bone and, thus, comprises one of the most relevant reasons for late treatment failure [4]. A 9-year follow-up study in patients with moderate to severe peri-implantitis revealed an onset of the disease within the first 3 years of function and a non-linear pattern of disease progression thereafter [5].

Since peri-implant mucositis commonly precedes peri-implantitis, the prevention of peri-implantitis is directed primarily toward the prevention of peri-implant mucositis [6]. For immediate recognition of developing inflammation routine, peri-implant monitoring has been recommended during peri-implant maintenance therapy (PIMT) [7]. Due to the partial analogy between periodontal and peri-implant diseases, the same diagnostic methods are commonly used for monitoring the health status of the peri-implant hard and soft tissues. According to recent recommendations, the soft tissue around implants should be probed periodically with gentle force [8]. Among other clinical and radiographic determinants, peri-implant mucositis is defined by the presence of bleeding on probing and a maximum pocket depth of 5 mm [9].

Taking account of the ultrastructural differences between periodontal and peri-implant tissue, there was a concern that probing depth measurements at implant sites show only poor reproducibility and diagnostic sensitivity [10]. In addition, probing might compromise the peri-implant epithelial attachment [11]. Moreover, it was suggested to use plastic probes in order to avoid damage to the titanium implant surface [12].

In fact, the treatment of dental implant with metal instruments induces changes of the chemical and physical properties within the titanium surface, thereby causing a considerable higher risk for bacterial adhesion and inflammation [13, 14]. Although the surface topography, particularly the surface roughness, does not exclusively determine the attractiveness for the adhesion and colonization of bacteria to titanium surfaces, several studies have reported a positive correlation showing less bacteria on smooth surfaces [15–17]. Animal studies in dogs, however, were not able to confirm the clinical impact of the surface roughness on the intensity of bacterial colonization and/or the strength of inflammation around dental implants [18, 19]. Treating titanium surfaces of dental implants with instruments made of less hard materials, i.e., plastic tips, causes some degree of abrasion (attrition) on the surface which ultimately results in impairment of the biocompatibility of the implant surface. Following to the treatment of rough implant surfaces, deposits of the carbon fiber instrument have been observed which interfered with the attachment of osteoblasts [20, 21].

From a clinical point of view, the use of a periodontal probe on a single occasion might leave only insignificant changes on the implant surface. Since PIMT including clinical monitoring of implants has been recommended at least twice per year [7], the potential surface changes might accumulate during many years of maintenance care and, finally, lead to an enhanced bacterial colonization and/or impaired attachment of host cells to the implant surface.

The present in vitro study aimed to determine if the motion of periodontal probes toward the surface of titanium implants causes changes within its ultrastructure. Moreover, the dependency of these changes on the material of the periodontal probe and the angulation of the probe should be evaluated.

## Materials and methods

### Study samples

Commercially available implants made of titanium alloy (Tissue Level Roxolid® Implant Ø 4,1mm, SLActive®, length 14.0 mm, Straumann AG, Basel, Switzerland) were selected for the experiments. Due to the dedicated transmucosal healing, these implants provided both smooth and rough surface areas. Each of the five implants was embedded in resin material (GC Bite Compound, GC EUROPE N V, Leuven, Belgium) onto a specially prepared sledge with four numbered sides allowing to define four experimental sections on each implant sample. The lateral borders of each experimental section have been marked at the insertion aid of the implant with a groove. All of the four experimental sections included the smooth machined surface (implant shoulder to the smooth/rough border) together with the cervical parts of the rough surface (smooth/rough border to the third implant thread), resulting in a total of 20 samples for each type (i.e., rough and smooth) of implant surface.

### Probes

The implants were instrumented with either a plastic- or metal-based probe. For plastic-based probing, a 0.2–0.25 N calibrated probe (Click-Probe, Kerr Corp, Orange, CA, USA) was used, while metal-based probing was performed using a WHO periodontal probe (DB765R, Aesculap, Tuttlingen, Germany), which is a titanium nitride coated steel probe also providing application force standardization (0.2 N). Force-standardized probings were chosen because measured probing depth around implants is very susceptible to force variation [22].

### Treatment of samples

**Fig. 1:**
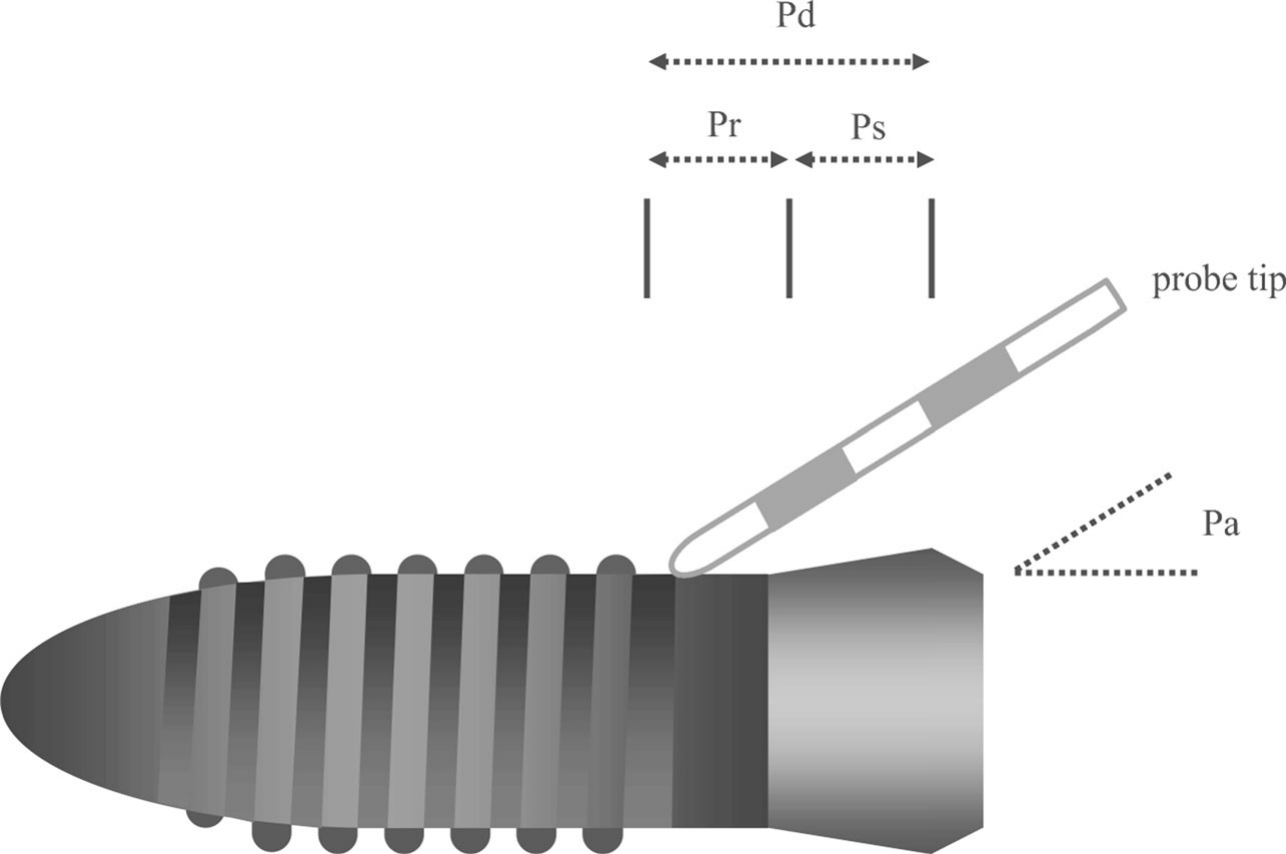
Experimental probing of implant surface: Pd, probing distance; Pr, probing distance on rough implant surface; Ps, probing distance on smooth implant surface; Pa, angulation of probe tip

To ensure continuous application force and standardized angulation while instrumenting the implants, the samples were clamped in a tripod during the experiment. Each probing motion was started within the smooth machined surface area of titanium (Ps) and continued to the third thread within the rough part of the surface (Pr) under calibrated force (0.2–0.25 N) and the appropriate angulation (Fig. 1). Between each probing motion, the sledge was laterally displaced approximately 1 mm to avoid the instrumentation of overlaid scratches. The application angle (Pa) of the instrument was set at 20° and 60°, measured as the angle between the implant axis and the tip of the probe. During experimental probing the respective angulation was adjusted using a contact goniometer which was fixed to the experimental set-up.

### Determination of implant surface roughness

The implant surface areas receiving experimental probing have been determined qualitatively and quantitatively before (baseline) and after treatment using a laser scanning microscope (LSM 800 MAT, Zeiss, Oberkochen, Germany) together with an image acquisition (ZEN) and an analysis software (ConfoMap Premium 7.4.8341, Zeiss, Oberkochen, Germany). The 2D profile roughness was determined as mean roughness (Ra), defined as the arithmetical mean of absolute height values Z(x), within a sampling length; as height of the highest peak (Rp); and as depth of the deepest valley (Rv) within a sampling length. In addition, the maximum height of profile  (peak-to-valley) Rt and mean roughness depth i.e. the deepest valley and the highest peak, within the evaluation length Rz were measured. For most parameters, a “parameter estimate” is calculated on each sampling length; these values are then averaged on the defined number of sampling lengths (ISO 4288:1996). Furthermore, a quantitative microscopic 3D analysis has been performed using various 3D parameters reflecting the surface roughness of untreated and treated samples. In particular the average deviations from a mean altitude value (arithmetic mean deviation of the surface; Sa) and the height between the highest peak and the mean plane (Sp) and depth between the mean plane and the deepest valley (Sv) have been determined along with the maximum height from the highest point to the deepest valley (Sz), defined as the sum of the largest peak height value and the largest pit depth value. Each profile and surface parameter has been determined prior and following to experimental probing on 5 samples (*n* = 5). For microscopic analysis a field of 0.36 to 0.5 mm (width) and 1.0 to 1.5 mm (length) has been selected on the highest prominence of each experimental area. Pre- and post-treatment microscopic images have been digitally matched using the grooves at the insertion aid as reference to ensure that identical parts of the experimental area have been analyzed prior and following to experimental probing.

### Statistical analysis

Sample size calculation was performed using the G Power Calculator (version 3.1) under the assumption (1) that plastic probes induce the least changes on titanium surfaces as compared with metal probes and (2) of an average effect size of d = 2.58 which has been calculated based on the mean Ra values on buccal and palatal aspects (valleys and threads) on control implants and implant surfaces treated with plastic instruments as reported by Cha et al. [13]. To reach a power of 0.95, the minimum sample size was 5 accordingly. All data are given as means (±SD). The datasets of each experimental group have been tested for homogeneity of variances using the Levene test and for normal distribution using the Kolmogorov-Smirnov test. Differences of profile and surface parameters prior and following to the application of the probe have been analyzed separately within each experimental group using paired *t*-test. Comparison of roughness parameters between groups following experimental probing of surfaces under different angulations has been done with two-sample *t*-test. Where appropriate (comparison of two groups), test procedures were two-tailed. For all test procedures, *p* values < 0.05 have been considered nominally significant. Correction for multiple testing using the Bonferroni procedure has been applied, setting the level of significance for comparisons between the four experimental groups (i.e., metal, plastic, angulation 20°, angulation 40°) at *p* = 0.0125. For all statistical procedures, SPSS software version 25.0 (SPSS Inc., Chicago, IL, USA) has been used.

## Results

### Qualitative surface assessment

#### Smooth surfaces

**Fig. 2:**
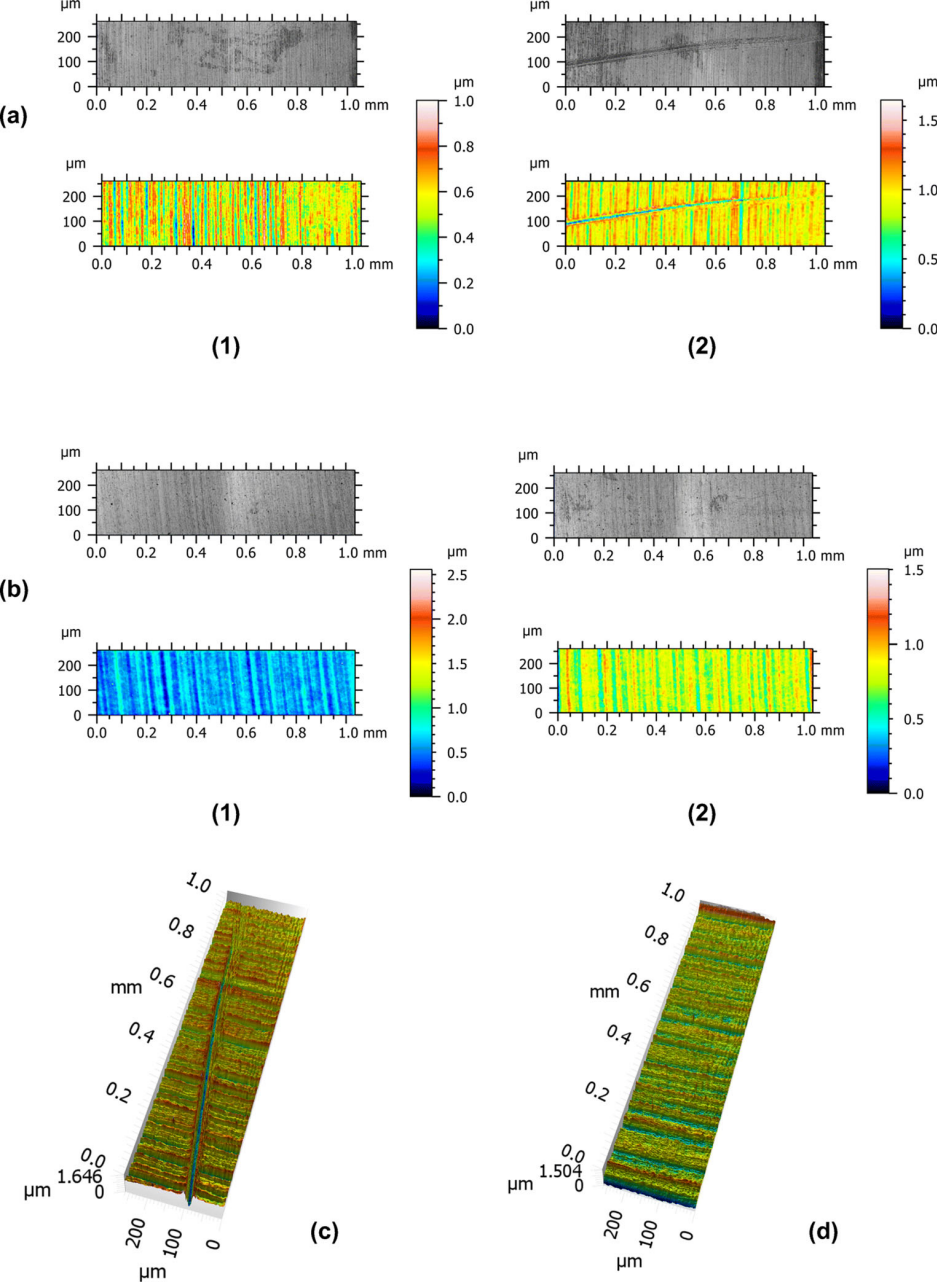
Microscopic image and topography of smooth implant surface. Two-dimensional view of sample treated with metal probe (**a**) and plastic probe (**b**) before (1) and following (2) to experimental probing. Three-dimensional view of sample treated with metal probe (**c**) and plastic probe (**d**)

The smooth surface areas of all samples show homogenous parallel running grooves compatible with machined implant surfaces. Comparing 2D microscopic images of the smooth surface areas, the metal probe obviously induced perpendicular running considerably more accentuated grooves whereas no changes are visible for plastic probes. This observation was confirmed also by the 3D microscopic images again showing grooves together with slightly elevated edges (Fig. 2).

#### Rough surfaces

**Fig. 3:**
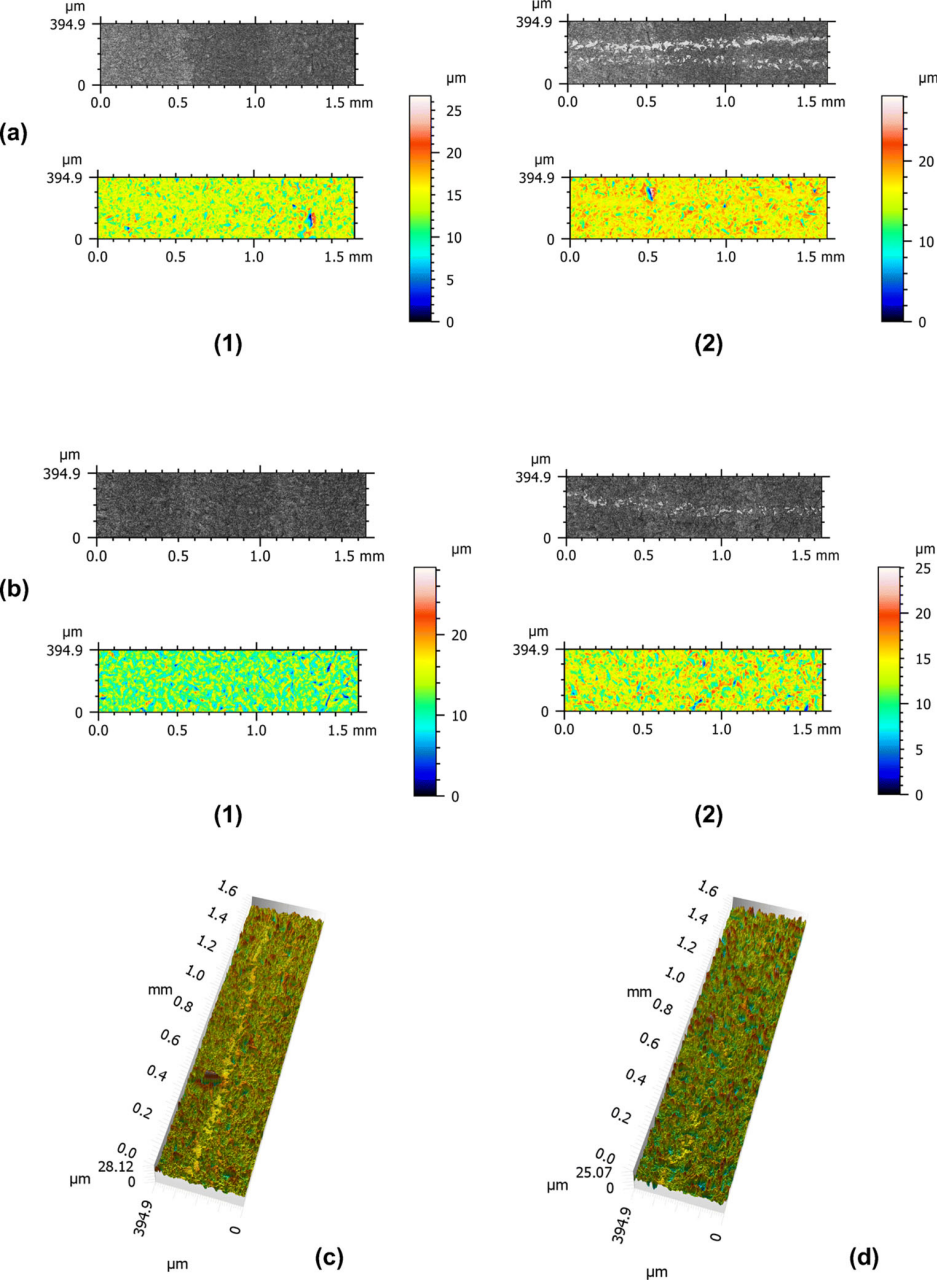
Microscopic image and topography of rough implant surface. Two-dimensional view of sample treated with metal probe (**a**) and plastic probe (**b**) before (1) and following (2) to experimental probing. Three-dimensional view of sample treated with metal probe (**c**) and plastic probe (**d**)

The rough parts of hydrophilic sandblasted and acid-etched titanium dental implants which were treated by a metal probe showed already visual contrasts between the matt reference and the light reflecting processed surfaces. When comparing the 2D microscopic images, the probed areas appeared more accentuated when using a metal probe than a plastic probe. There were no observed crumbs of the probe material on any sample. Considering the 3D microscopic images, the lines resemble gutters with smooth bottom and raised boundary which are more distinct for samples treated by a metal probe than by a plastic probe (Fig. 3).

### Laser scanning microscope assessment of surface roughness

#### Metal probe

The average profile roughness (Ra) on smooth surfaces prior to experimental probing increased from 0.087 (± 0.023) μm to 0.104 μm (± 0.034) (20°) (*p* = 0.150) and from 0.091 (± 0.017) μm to 0.133 μm (± 0.061) (60°) (*p* = 0.205). When considering rough surfaces, the average roughness (Ra) shows a slight but not significant decrease using an application angle of 20° from 2.078 (± 0.410) μm to 2.044 (± 0.338) μm (*p* = 0.664) and a slight increase (*p* = 0.494) from 2.144 (± 0.306) μm to 2.228 (± 0.267) μm using an application angle of 60° which did also not reach statistical significance (Table 1). Considering smooth surface areas, Rp was higher for metal probes used with an angle of 20° (*p* = 0.007). Also Rv (20°, *p* = 0.016; 60°, *p* = 0.043) and Rt (20°, *p* = 0.026) are nominally significantly elevated on smooth surfaces. However, this association remained not significant after correction for multiple testing. On previously rough surfaces, Rp (*p* = 0.020), Rz (*p* = 0.054), and Rt (*p* = 0.024) were also nominally significant when probing was applied at an angulation at 20°, but, again, the differences did not reach significance after Bonferroni correction. The 3D parameters are elevated on smooth surfaces and decreased on rough parts of the implant, but these changes did not reach significance.

**Table 1:** Mean of 2D and 3D roughness parameter as obtained with laser scanning microscopy on samples of titanium implants prior and following to the application of a metal probe

	Angulation 20°			Angulation 60°		
	Before probing (μm ± SD)	After probing (μm ± SD)	*p* Value	Before probing (μm ± SD)	After probing (μm ± SD)	*p* Value
Smooth surface						
Ra	0.087 (± 0.023)	0.104 (± 0.034)	*p* = 0.150	0.091 (± 0.017)	0.133 (± 0.061)	*p* = 0.205
Rp	0.250 (± 0.048)	0.373 (± 0.097)	*p* *= 0.007*	0.283 (± 0.051)	0.413 (± 0.159)	*p* = 0.144
Rv	0.285 (± 0.060)	0.446 (± 0.125)	*p* = 0.016	0.269 (± 0.044)	0.512 (± 0.174)	*p* = 0.043
Rz	0.535 (± 0.104)	0.613 (± 0.299)	*p* = 0.640	0.552 (± 0.093)	0.658 (± 0.296)	*p* = 0.463
Rt	0.689 (± 0.173)	0.931 (± 0.224)	*p* = 0.026	0.712 (± 0.161)	0.838 (± 0.320)	*p* = 0.505
Sa	0.090 (± 0.024)	0.100 (± 0.024)	*p* = 0.099	0.099 (± 0.018)	0.109 (± 0.020)	*p* = 0.178
Sp	1.095 (± 1.025)	0.814 (± 0.357)	*p* = 0.412	1.14 (± 0.970)	1.044 (± 0.386)	*p* = 0.834
Sv	0.579 (± 0.105)	0.696 (± 0.112)	*p* = 0.123	0.481 (± 0.077)	1.00 (± 0.688)	*p* = 0.161
Sz	1.672 (± 1.076)	1.512 (± 0.345)	*p* = 0.662	1.623 (± 0.943)	2.044 (± 1.032)	*p* = 0.445
Rough surface						
Ra	2.078 (± 0.410)	2.044 (± 0.338)	*p* = 0.664	2.144 (± 0.306)	2.228 (± 0.267)	*p* = 0.494
Rp	7.394 (± 1.217)	5.916 (± 0.804)	*p* = 0.020	7.284 (± 1.194)	6.378 (± 0.804)	*p* = 0.173
Rv	7.842 (± 1.173)	7.274 (± 0.891)	*p* = 0.237	8.066 (± 1.007)	7.636 (± 0.470)	*p* = 0.350
Rz	15.240 (± 2.241)	13.200 (± 1.649)	*p* = 0.054	15.360 (± 1.851)	14.020 (± 1.238)	*p* = 0.137
Rt	22.480 (± 3.687)	17.240 (± 1.387)	*p* = 0.024	25.280 (± 8.671)	19.720 (± 2.100)	*p* = 0.242
Sa	2.206 (± 0.395)	2.146 (± 0.301)	*p* = 0.463	2.222 (± 0.372)	2.282 (± 0.246)	*p* = 0.649
Sp	14.720 (± 3.419)	12.100 (± 1.037)	*p* = 0.159	15.300 (± 4.867)	16.620 (± 3.477)	*p* = 0.693
Sv	17.160 (± 6.341)	15.040 (± 1.532)	*p* = 0.565	17.360 (± 5.445)	15.500 (± 1.198)	*p* = 0.447
Sz	31.860 (± 9.384)	27.140 (± 0.744)	*p* = 0.342	32.680 (± 9.579)	32.120 (± 4.544)	*p* = 0.909

Differences have been analyzed using the paired *t*-test; SD, standard deviation, italic style indicates significant p-values following to Bonferroni correction for multiple testing.

#### Plastic probe

Using plastic probes on smooth implant surfaces, the roughness parameters remained virtually unchanged comparing the surface topography prior and following to the experimental probing irrespective of the application angle (Table 2). On rough surfaces a small increase of some surface parameters following to probe application at 20° was observed. The opposite trend was found at 60° showing a slight decrease of surface roughness after probing. None of these differences reached statistical significance.

**Table 2:** Mean of 2D and 3D roughness parameter as obtained with laser scanning microscopy on samples of titanium implants prior and following to the application of a plastic probe

	Angulation 20°			Angulation 60°		
	Before probing (μm ± SD)	After probing (μm ± SD)	*p* Value	Before probing (μm ± SD)	After probing (μm ± SD)	*p* Value
Smooth surface						
Ra	0.092 (± 0.020)	0.091 (± 0.015)	*p* = 0.643	0.086 (± 0.020)	0.087 (± 0.018)	*p* = 0.863
Rp	0.270 (± 0.041)	0.281 (± 0.039)	*p* = 0.238	0.254 (± 0.069)	0.261 (± 0.029)	*p* = 0.770
Rv	0.311 (± 0.047)	0.301 (± 0.059)	*p* = 0.551	0.271 (± 0.047)	0.282 (± 0.064)	*p* = 0.261
Rz	0.581 (± 0.086)	0.582 (± 0.098)	*p* = 0.944	0.525 (± 0.110)	0.543 (± 0.091)	*p* = 0.472
Rt	0.765 (± 0.128)	0.722 (± 0.150)	*p* = 0.280	0.650 (± 0.168)	0.610 (± 0.093)	*p* = 0.461
Sa	0.100 (± 0.016)	0.095 (± 0.016)	*p* = 0.140	0.098 (± 0.023)	0.095 (± 0.026)	*p* = 0.285
Sp	1.304 (± 0.948)	0.562 (± 0.125)	*p* = 0.170	0.765 (± 0.169)	0.565 (± 0.113)	*p* = 0.054
Sv	0.538 (± 0.100)	0.555 (± 0.119)	*p* = 0.736	0.586 (± 0.131)	0.580 (± 0.220)	*p* = 0.917
Sz	1.844 (± 0.991)	1.12 (± 0.202)	*p* = 0.195	1.35 (± 0.291)	1.15 (± 0.314)	*p* = 0.075
Rough surface						
Ra	2.104 (± 0.207)	2.354 (± 0.286)	*p* = 0.105	2.260 (± 0.358)	2.206 (± 0.235)	*p* = 0.722
Rp	7.236 (± 0.681)	7.842 (± 1.640)	*p* = 0.525	7.894 (± 1.183)	6.624 (± 1.563)	*p* = 0.262
Rv	7.678 (± 0.687)	8.174 (± 0.789)	*p* = 0.202	6.320 (± 3.155)	7.710 (± 0.998)	*p* = 0.460
Rz	14.920 (± 1.201)	16.020 (± 1.851)	*p* = 0.214	16.320 (± 3.073)	14.320 (± 1.914)	*p* = 0.203
Rt	21.460 (± 2.953)	22.280 (± 3.983)	*p* = 0.719	24.160 (± 6.802)	19.280 (± 2.181)	*p* = 0.182
Sa	2.218 (± 0.269)	2.282 (± 0.291)	*p* = 0.336	2.360 (± 0.328)	2.224 (± 0.179)	*p* = 0.248
Sp	15.080 (± 1.912)	16.500 (± 4.691)	*p* = 0.559	16.260 (± 3.205)	16.180 (± 4.334)	*p* = 0.974
Sv	14.500 (± 3.455)	14.280 (± 1.869)	*p* = 0.908	15.100 (± 3.433)	15.660 (± 3.463)	*p* = 0.613
Sz	29.540 (± 4.997)	30.740 (± 5.847)	p = 0.770	31.360 (± 6.097)	31.820 (± 7.382)	*p* = 0.872

Differences have been analyzed using the paired *t*-test; SD, standard deviation

### Changes of surface roughness depending on the application angle and the probe material

Comparing the profile and surface roughness after experimental probing with different angulations revealed opposite trends for both types of materials. For metal probes the surface roughness was positively associated with the angulation, whereas plastic probes induced a slightly reduced surface roughness at 60° as compared with 20°. However, the differences between angulations did not reach significance for metal and plastic probes (Table 3).

**Table 3:** Comparison of differences of the mean of various 2D and 3D roughness parameters on titanium implants following to the application of a metal and plastic probe under an angulation of 20° and 60°

	Metal probe		Plastic probe	
	Smooth surface (20° vs. 60°)	Rough surface (20° vs. 60°)	Smooth surface (20° vs. 60°)	Rough surface (20° vs. 60°)
Ra	*p* = 0.367	*p* = 0.367	*p* = 0.746	*p* = 0.397
Rp	*p* = 0.646	*p* = 0.390	*p* = 0.393	*p* = 0.264
Rv	*p* = 0.512	*p* = 0.445	*p* = 0.627	*p* = 0.438
Rz	*p* = 0.818	*p* = 0.400	*p* = 0.532	*p* = 0.191
Rt	*p* = 0.609	*p* = 0.059	*p* = 0.191	*p* = 0.178
Sa	*p* = 0.536	*p* = 0.457	*p* = 0.997	*p* = 0.714
Sp	*p* = 0.357	*p* = 0.041	*p* = 0.967	*p* = 0.914
Sv	*p* = 0.358	*p* = 0.611	*p* = 0.827	*p* = 0.456
Sz	*p* = 0.306	*p* = 0.070	*p* = 0.871	*p* = 0.804

Differences have been analyzed using the two-sample *t*-test; SD, standard deviation

## Discussion

For monitoring of implant health and prevention of infection of the peri-implant tissue, routine maintenance care including probing of the peri-implant tissue has been recommended [7]. Currently, the overall clinical significance of routine clinical probing of the peri-implant tissue has been challenged due to different reasons specifically belonging to the reproducibility and sensitivity to detect peri-implant disease [10, 23]. For example, the determination of the peri-implant pocket depth seems particularly difficult at implants with platform switching and/or expanding emergence profile [24]. On the contrary peri-implant probing seems to be indispensable in monitoring and maintaining peri-implant health [8]. Yet, there exists only limited evidence on the changes of the implant surface associated with routine probing.

Apart from qualitative assessment of ultrastructural changes of the implant surface, various parameters representing the physical roughness are commonly used to quantitatively determine these changes [25, 26]. Together with the surface free energy, particularly the surface roughness has been proposed as the major determining factor on the micrometer scale for the retention of bacteria to the implant surface [27]. Comparing the impact of both determinants, the surface roughness seems more important toward the surface free energy [28, 29].

Mostly, two groups of instruments that are based on tactile or optical determination of the surface texture are used for the measurement of the surface roughness [30]. In addition to the non-contact evaluation of the surface texture, the optical methods have the advantage to analyze not only linear (2D) profiles but also areal (3D) surface parameters. A wide number of methods are available for optical analysis of the surface texture among which the confocal microscopy allows for high vertical and horizontal resolution [30, 31].

Herein, topographical 2D and 3D parameters have been determined using confocal laser scanning microscopy. Considering the various 2D amplitude parameters for untreated implant surfaces, Ra was approximately 0.10 μm on smooth surfaces and 2.0 μm on rough implant surfaces. These values are in line with previous studies on the surface roughness of the same implant type [26, 32], thus confirming the high reproducibility and reliability of the confocal laser scanning microscope for the quantitative determination of ultrastructural changes of the titanium surface receiving experimental probing.

The present results revealed that the motion of the probe tip across the implant body leads to very discrete surface changes only. The metal probe slightly increased the roughness on the smooth surface areas and left a trend for decreased roughness at previously rough parts of the implant. Even smaller changes were observed for surfaces following to the contact with plastic probes. None of these changes reached statistical significance.

So far, there exists a considerable controversy on the importance of the implant surface roughness on bacterial adhesion. Several studies reported a positive correlation between bacterial adhesion and/or the rate of plaque formation with the surface roughness in vitro [33–35] and a shift toward a dysbiotic microflora together with a higher rate of inflammation around implant abutments with rougher surface topography in vivo [17, 36, 37]. On the contrary, the surface roughness did not influence the adhesion of *Streptococcus epidermidis* and *Staphylococcus sanguinis* on titanium implants [25] and had also no influence on the development of plaque and peri-implant inflammation [18, 38]. The lack of consensus on the influence of surface roughness on bacterial adhesion might be attributed to the confinement on two global roughness parameters, i.e., the average roughness (Ra) and the root mean square roughness (Rrms), for the quantitative characterization of the surface topography of dental implants in most of these studies [26, 39]. Since both parameters give no information on the spatial distribution or particular shape of the single features of the ultrastructure, two surfaces might provide the same Ra and Rrms value despite considerable differences of their topography [40]. For a comprehensive analysis of the surface topography, a set of “S parameters” considering not only linear profiles but defined areas of the surface have been proposed instead [39]. Herein, also the 3D analysis revealed only minor and not significant differences of the surface roughness before and after the application of metal probes and even less with plastic probes.

On smooth implant surfaces, one parameter, i.e., Rp, that represents extreme values for the heights and depths of the surface structure was significantly elevated after treatment with metal probes [42]. This observation might indicate that the movement of the probe tip across the implant surface causes rather linear than areal changes, i.e., scratches, at a small number of sites of the surface profile. In fact, the qualitative microscopic analysis confirms linear-shaped scratches caused by the movement of the probe tip.

Different from smooth implant surfaces, the rough surface areas are dedicated to be colonized by the host cells, particularly osteoblasts, in order to integrate the implant body into the osseous tissue. Compared with the smooth parts of the implant surface, the rough surface should be enclosed entirely into the alveolar bone. The rough surface parts of the implant yet might gain contact with the periodontal probe, if peri-implant disease has already caused partial disintegration of the implant [43]. Herein, the rough surface areas showed a tendency for reduced roughness values following experimental probing. As found in the qualitative microscopic analysis, the previous surface structure has been flattened within these scratches. According to recent reports, the adhesion, proliferation, and differentiation of osteoblasts are strongly dependent upon the roughness of titanium surfaces. Rougher surfaces seem to attract osteoblasts more effectively as compared with machined areas or surfaces showing only minor roughness [44, 45]. Hence, the equalization of the surface topography due to routine probing might interfere with the reattachment of osteoblasts following to the successful treatment of peri-implant defects.

Previous studies on periodontal instruments, i.e., ultrasonic scaler and air-powder devices, have found a strong correlation between the roughness of root surfaces and the angulation of the working tip [46, 47]. It is commonly recommended to align the probe as parallel as possible at low angulations related to the implant surface. Considering the lower diameter of implants in comparison with natural teeth together with the mostly protruding emergence profile of the prosthetic restoration, one should realize that probing is commonly performed under higher angulations, i.e., 20°, in daily practice. In order to be able to determine an inherent influence of the application angle on changes of the implant surface, herein, the higher angulation was set at 60° which might be reached clinically only rarely. Comparing the changes of the implant surface topography according to the application angle revealed no differences following to the contact with metal and plastic probes.

From a clinical perspective, yet, it seems questionable if the minor changes of the surface topography as found in this study might, anyhow, impair the peri-implant conditions and/or even increase the risk for the manifestation of a significant bacterial infection ultimately increasing peri-implant inflammation. This study did not determine the influence of the surface changes on the ability of bacteria to adhere to the implant surface.

The present results were found under experimental conditions which might not entirely reflect the real clinical situation during probing of the peri-implant tissue. In this context specifically the individual implant design, i.e., the shape of the implant, might have a considerable impact on the alignment and proper application of the periodontal probe. In addition, in the current study, the application force was adjusted from 0.2 to 0.25 N. Due to the placement into the peri-implant pocket, the probe might be exposed to even higher vectorial application forces in the clinical situation which are primarily dependent on the strength of the peri-implant soft tissue. Moreover, this study considered only one specific type of rough implant surface. The considerable morphological differences between various types of rough implant surfaces might lead to differences in surface changes associated with routine probing including the potential for abrasion of the probe material. Yet, considering the mostly slight but partially stronger ultrastructural changes of the implant surfaces following to contact with metal probes, the use of plastic probes for the clinical evaluation of the peri-implant tissue seems preferable so far.

Taken together this in vitro study has shown that the movement of metal probes and to a lesser extent also of plastic probes over the implant surface caused discrete changes on both smooth and rough titanium surfaces. However, it remains to be elucidated if these changes might gain clinical relevance.
