# Correction to: Ultrastructural changes of smooth and rough titanium implant surfaces induced by metal and plastic periodontal probes

**DOI:** 10.1007/s00784-021-04216-9

**Published:** 2021-11-18

**Authors:** Matthias Folwaczny, Torsten Rudolf, Iris Frasheri, Madlena Betthäuser

**Affiliations:** 1 Department of Conservative Dentistry and Periodontology, University Hospital, Ludwig-Maximilians-University, Goethestr. 70, Munich, 80336 Germany; 2 ZEISSMicroscopy Customer Center Europe, Carl ZeissMicroscopy GmbH, Oberkochen, Germany

Correction to: Clinical Oral Investigations

https://doi.org/10.1007/s00784-020–03341-1

The article “Ultrastructural changes of smooth and rough titanium implant surfaces induced by metal and plastic periodontal probes”, written by Matthias Folwaczny, Torsten Rudolf, Iris Frasheri and Madlena Betthäuser, was originally published Online First without Open Access. After publication in volume 25, issue 1, page 105–114 the author decided to opt for Open Choice and to make the article an Open Access publication. Therefore, the copyright of the article has been changed to © The Author(s) 2020 and the article is forthwith distributed under the terms of the Creative Commons Attribution 4.0 International License, which permits use, sharing, adaptation, distribution and reproduction in any medium or format, as long as you give appropriate credit to the original author(s) and the source, provide a link to the Creative Commons license, and indicate if changes were made. The images or other third party material in this article are included in the article’s Creative Commons license, unless indicated otherwise in a credit line to the material. If material is not included in the article’s Creative Commons license and your intended use is not permitted by statutory regulation or exceeds the permitted use, you will need to obtain permission directly from the copyright holder. To view a copy of this license, visit http://creativecommons.org/licenses/by/4.0.

The original article has been corrected.

